# A Novel Hierarchical Template Matching Model for Cardiac Motion Estimation

**DOI:** 10.1038/s41598-018-22543-y

**Published:** 2018-03-14

**Authors:** Jayendra M. Bhalodiya, Arnab Palit, Manoj K. Tiwari, Sanjay K. Prasad, Sunil K. Bhudia, Theodoros N. Arvanitis, Mark A. Williams

**Affiliations:** 1 Warwick Manufacturing Group (WMG), University of Warwick, CV4 7AL Coventry, United Kingdom; 2 Indian Institute of Technology Kharagpur, Kharagpur, West Bengal India; 3 Royal Brompton and Harefield NHS Foundation Trust, London, United Kingdom; 4 Institute of Digital Healthcare, WMG, University of Warwick, Coventry, United Kingdom

## Abstract

Cardiovascular disease diagnosis and prognosis can be improved by measuring patient-specific *in-vivo* local myocardial strain using Magnetic Resonance Imaging. Local myocardial strain can be determined by tracking the movement of sample muscles points during cardiac cycle using cardiac motion estimation model. The tracking accuracy of the benchmark Free Form Deformation (FFD) model is greatly affected due to its dependency on tunable parameters and regularisation function. Therefore, Hierarchical Template Matching (HTM) model, which is independent of tunable parameters, regularisation function, and image-specific features, is proposed in this article. HTM has dense and uniform points correspondence that provides HTM with the ability to estimate local muscular deformation with a promising accuracy of less than half a millimetre of cardiac wall muscle. As a result, the muscles tracking accuracy has been significantly (p < 0.001) improved (30%) compared to the benchmark model. Such merits of HTM provide reliably calculated clinical measures which can be incorporated into the decision-making process of cardiac disease diagnosis and prognosis.

## Introduction

Cardiovascular Diseases (CVDs) are amongst the leading causes of death globally^1^. The purpose of cardiac image analysis is to provide tools for disease diagnosis and prognosis. The structural analysis such as muscular strain (shortening or lengthening of muscles) has increased research attention compared to global analysis like blood ejection fraction^2,3^. Cardiac Motion Estimation^4–7^ (CME) with gold standard^8,9^ Magnetic Resonance Imaging (MRI) can be used to calculate subject-specific muscular strain of the myocardium (heart wall)^4,10^. The subject-specific^11^ or importantly patient-specific muscular strain could be beneficial for the treatment of cardiac arrhythmia^12^, ischemia^13^, cardiomyopathy^14^, valve diseases^15^ and chemotherapy^16^. For example, Cardiac Resynchronization Therapy (CRT) with a pacemaker implant is one of the common clinical practices in patients with heart failure to maintain the mechanical movement of the heart. In clinical practice, the effectiveness of CRT is based on echocardiographic indices. However, researchers have reported serious dyssynchrony even after months of pacemaker implant^17^. Echocardiography, as a result, is not recommended as per current clinical guidelines^12^. An innovative use of muscular strain would be to target specific regions of the heart for placing the lead (wire-end of pacemaker device)^12,17^ in patients requiring CRT. Concisely, the patient-specific cardiac disease treatment could be improved by strain value-based disease diagnosis and prognosis^18^, and strain values could be reliably derived using accurate cardiac motion estimation model^4–6^.

A typical cardiac motion can be estimated by tracking sample myocardium muscles points throughout all the images of a cardiac cycle. The myocardium muscles can be tagged while developing MRI image, and these tag points could be used as sample points to track the detailed movement of the myocardium wall during a cardiac cycle. In the past decades, researchers have developed various CME models to measure cardiac motion and consequently, the ventricular wall strain. The state-of-the-art CME models are Optical Flow^19,20^ (OF), Harmonic Phase^4^ (HARP), and Free Form Deformation^10,21,22^ (FFD). OF and its extensions are independent of tag appearance but are extremely sensitive to the signal to noise ratio, and therefore, they fail to track muscles in low-resolution images^23^. HARP is based on Fourier domain analysis and capable of tracking arbitrary points of the image. However, it is natively low dimensional, highly sensitive to noise, underestimates motion because of aliasing artefacts and inaccurate for myocardial borderline muscles^23–25^. Non-rigid image registration of FFD is based on B-spline functions and performs better than OF and HARP^23,24^. The fast-FFD has been proposed with a strategy of concurrently optimising control points^26^.

However, FFD has many tunable parameters such as (i) grid spacing in x and y directions, (ii) multiple grid levels, (iii) similarity measure, (iv) regularisation function, (v) a combination of different regularisation functions, and (vi) the maximum number of iterations. Therefore, the accuracy of the FFD greatly depends on the proper selection of these parameters for a particular pair of images. Additionally, the values of parameters may differ for a different set of image pairs. As a result, a fixed set of parameter values does not provide same accurate output for all the image pairs in a cardiac cycle. For example, the parameter values used with images A and B may not produce an accurate output for a different image pair C and D, as the best parameter values for image pair C and D might be different as used for image pair A and B. A typical cardiac motion can be recorded with 19 images (may vary as per subject) during a cardiac cycle, which is 18 image pairs. FFD output does not remain accurate for all 18 pairs of the cardiac cycle, and the inaccurate output leads to incorrect clinical measures. Contrary to FFD behaviour and according to a recent clinical contribution, the whole cardiac cycle strain values are crucial clinical measures^18^. Therefore, a CME model that remains accurate during the whole cardiac cycle is essential.

In addition, the FFD has smoothing effect which eventually underestimates the radial strain^27^. Incorporating cine MRI with tag MRI can improve the calculation of radial strain^24^, but this will incorporate additional dependency on cine imaging. Moreover, the spatial and temporal alignment between cine and tag MRI will introduce additional error in the strain estimation. Tag points are sparse in the radial direction of heart vessel, which increases the difficulty for accurate radial strain measurement in heart muscles^27^. MRI images suffer from fading of tag points due to blood flow in vessels, and therefore, it is difficult to track the tag points. Hence, a CME model, which is less dependent or independent of tag points, is expected.

Overall, existing benchmark model FFD has limited accuracy due to the dependency on tunable parameters and regularisation function which result into incorrect clinical measures^23–25,27–29^.

In this paper, a novel Hierarchical Template Matching (HTM) model is proposed which is independent of tunable parameters, tag intersection points and regularisation function. The HTM considers the image as a set of points and is the core part of CME framework that estimates the muscles points displacement during the cardiac cycle. HTM algorithm consists of three main steps: (i) selecting image points (ii) establishing point-correspondence between multiple images, and (iii) calculating geometric transformation among them. Points are dense, uniformly distributed, and automatically derived with hierarchical normalised cross-correlation and correlation-coefficient, which are proven as optimal matching criteria^30–32^. The geometric transformation has been calculated with Local Weighted-Mean^33–36^ (LWM). LWM is capable of calculating local image area-based non-linear transformation. LWM has provided promising results for the transformation of satellite images^37,38^. The displacement vectors of points have been used to calculate deformation followed by circumferential and radial strain^39^.

Section 2 includes the proposed HTM model and strain calculation steps. The results, validation approach and calculated strain values for a given MRI data set have been reported in Section 3. The discussion of clinical impact has been mentioned in Section 4, followed by a conclusion in Section 5.

## Methods

**Figure 1:**
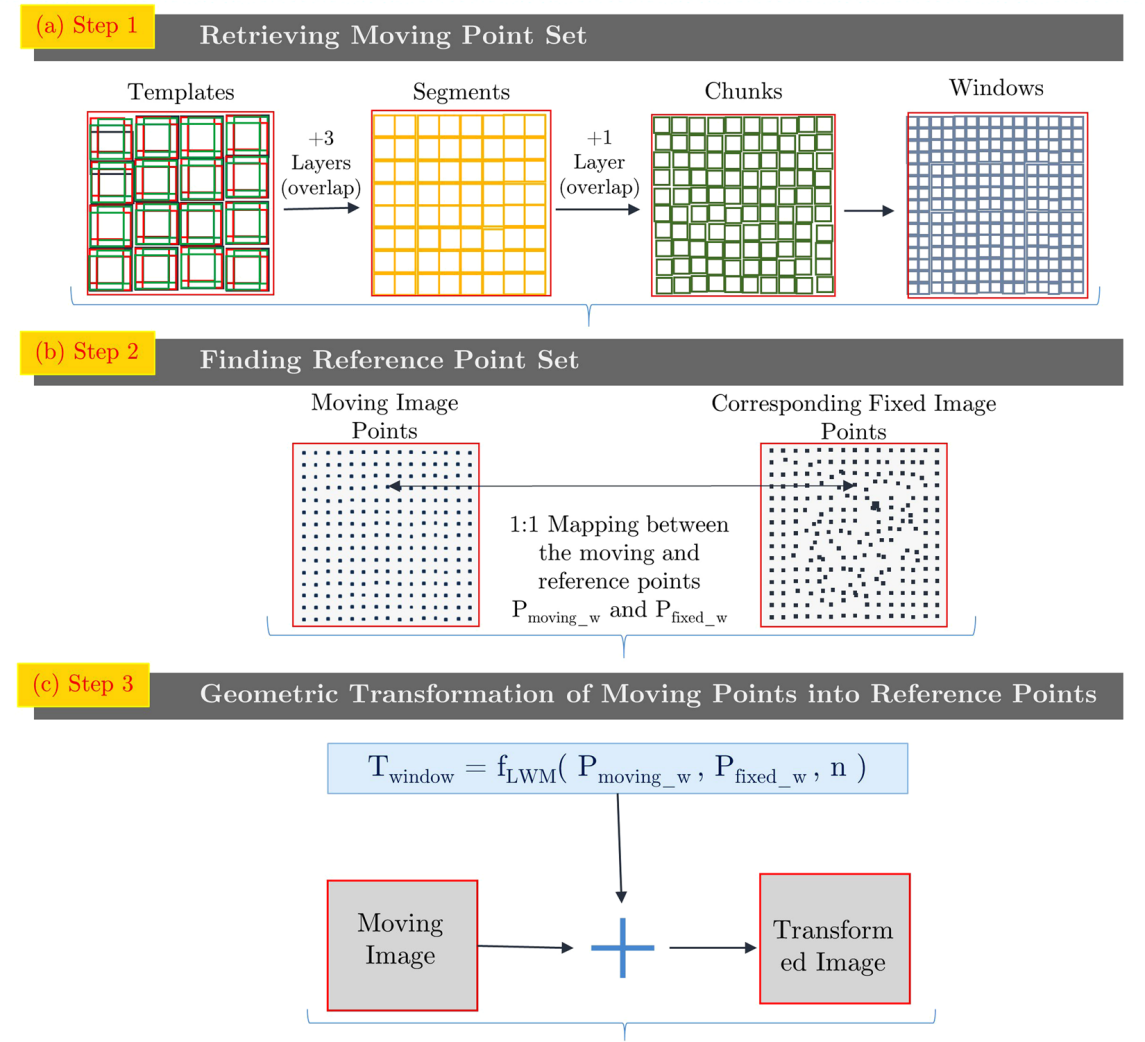
Overview of HTM model. These steps are repeated for all images pairs of the cardiac cycle to estimate strain in the myocardial muscles as a sequence of geometric transformation between image pairs.

**Figure 2:**
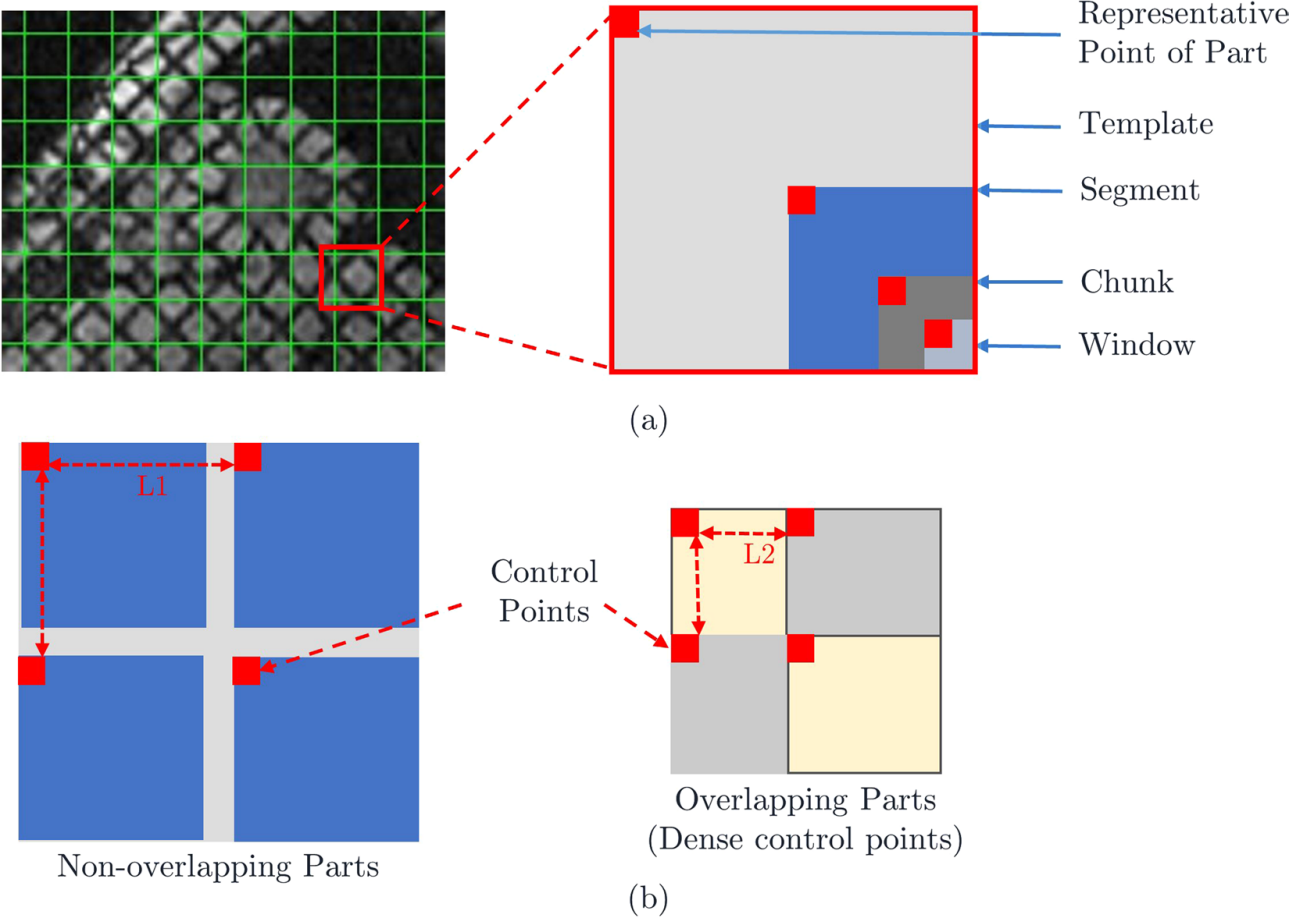
(**a**) Pictorial definitions of Template, Segment, Chunk, Window, Representative or Control Point. (**b**) Structure of overlapping parts layer to derive dense control points.

The Hierarchical Template Matching (HTM)-based non-rigid image registration algorithm has been proposed, and the cardiac motion has been estimated by a sequence of image registrations over the cardiac cycle. As mentioned in Fig. 1, HTM consists of three steps: (i) Retrieving moving image point set (ii) Finding corresponding reference image point set (iii) Calculating geometric transformation between moving and the reference image point set. These three steps are performed with all the image pairs of the cardiac cycle to estimate the cardiac motion and strain values, which is described in section 2.4. Template, Segment, Chunk and Window are defined in Fig. 2, and the word ‘part’ is used for any of these four words. The phrase ‘target sliding region’ is used for the reference image area.

### Retrieving Moving Point Set

The moving image has been divided into t × t size image areas, which are Templates. Templates are divided into t/2 × t/2 size Segments, and Segments are divided into t/4 × t/4 size Chunks. Finally, Chunks are divided into t/8 × t/8 size Windows. The initial size of t is set as 16. Therefore the sizes of parts are as, Template 16 × 16, Segment 8 × 8, Chunk 4 × 4, and Window 2 × 2.

The first point of all the parts is stored in a separate set of points, which is a uniformly distributed point set, M = {m_1_, m_2_, …, m_n_}. The point set made up of all Window points is uniformly distributed as well as dense P_moving_ = {p_1_, p_2_, …, p_n_}, which is a moving point set.

### Finding Reference Point Set

The hierarchical structure has been used to identify the reference image points corresponding to the moving image points. The proposed hierarchical structure has two crucial components: template matching and overlapping layers. Template matching has been performed using Normalized cross-correlation (NCC) and correlation coefficient (CC). NCC takes two images as input and gives an output matrix of CC. The output matrix contains values ranging from −1.0 to +1.0. The maximum matrix value indicates the expected location of matching between both images. We have adopted NCC^40,41^ as a three-step procedure:

i. Select moving image part and compute its cross-correlation with corresponding reference image sliding area.
ii. Evaluate local sums by pre-calculating running sums^41^.
iii.  Apply local sums to normalise the cross-correlation values to calculate CC.

As shown in Fig. 1 Step1, three overlapping layers are designed between Template and Segment layer, and one layer is designed between Segment and Chunk layer. The three overlapping layers have sizes 14 × 14, 12 × 12, 10 × 10, whereas the one layer between Segment and Chunk is of size 6 × 6. The overlapping layers perform the crucial task of improving accuracy and reducing the size of reference image area during hierarchical matching. The mathematical definition of NCC is mentioned in Equation 1.

1 $$\gamma (u,v)=\frac{\sum _{x,y}[f(x,y)-{\bar{f}}_{u,v}][p(x-u,y-v)-\overline{p}]}{{\{\sum _{x,y}{[f({\rm{x}},{\rm{y}})-{\bar{f}}_{u,v}]}^{2}\sum _{x,y}{[p({\rm{x}}-{\rm{u}},{\rm{y}}-{\rm{v}})-\overline{p}]}^{2}\}}^{0.5}}$$

where *f* is the reference image, $\overline{p}$ is mean of moving image template, ${\overline{f}}_{u,v}$ is mean of *f* (x, y) that is reference image area under moving image template. The maximum of *γ* (u, v) is used to calculate the location of matching reference image area.

Equation (2) represents the first step of hierarchical structure, which is NCC between reference image and template of moving image.

2 $${I}_{M}=\sum _{i=1}^{t}M{T}_{i}\phantom{\rule{5em}{0ex}}R{T}_{i}=NCC(M{T}_{i},{I}_{R})$$

where I_M_: Moving Image, I_R_: Reference Image, MT: Moving Template, RT: Reference Template i:i^th^ template, NCC: Normalized Cross-Correlation.

As mentioned in equation (3), the moving template section is used as an input for the overlapping layers, which perform NCC with the reference image template.

3 $$M{X}_{im}\subset M{T}_{i}\phantom{\rule{5em}{0ex}}R{X}_{im}=NCC({{\rm{MX}}}_{im},{{\rm{RT}}}_{i})$$

where the size of X is (st-2) × (st-2), st × st is the size of Template, MX: moving template section, RX: Reference template section, i: i^th^ template, m: m^th^ template section. The procedure of Equation (3) is performed with three different sizes of X, and the output RX_im_ is used as input for Equation (4).

As mentioned in Equation (4), matching reference template section (RX) has performed NCC with moving segment to find corresponding reference segment.

4 $$M{T}_{i}=\sum _{j=1}^{4}M{S}_{ij}\quad \quad \quad \quad R{S}_{ij}=NCC(M{S}_{ij},R{X}_{im})$$

where MS: Moving Segment, RS: Reference Segment, i: i^th^ template, m: m^th^ template section, j: j^th^ segment.

Equation (5) represents the overlapping layer between Segment and Chunk layer. The input for NCC is reference segment and moving segment section. It gives the reference segment section as an output.

5 $$M{Y}_{ijy}\subset M{S}_{ij}\quad \quad \quad \quad R{Y}_{ijy}=NCC({{\rm{MY}}}_{ijy},R{S}_{ij})$$

where MY: Moving segment section, RY: Reference segment section, i: i^th^ template, j: j^th^ segment, y: y^th^ segment section.

As mentioned in Equation (6), Matching reference segment section (RY) is further used as an input of NCC with moving chunk. It gives the matching reference image chunk.

6 $$M{S}_{ij}=\sum _{k=1}^{4}M{C}_{ijk}\quad \quad \quad \quad R{C}_{ijk}=NCC(M{C}_{ijk},R{Y}_{ijy})$$

where MC: Moving Chunk, RC: Reference Chunk, i: i^th^ template, j: j^th^ segment, k: k^th^ chunk, y: y^th^ segment section.

As mentioned in Equation (7), as a final step, NCC between reference image chunk and moving image window is performed. It provides the matching reference image window corresponding to the moving image window.

7 $$M{C}_{ijk}=\sum _{l=1}^{4}M{W}_{ijkl}\quad \quad \quad \quad R{W}_{ijkl}=NCC(M{W}_{ijkl},R{C}_{ijk})$$

where MW: Moving Window, RW: Reference Window, i: i^th^ template, j: j^th^ segment, k: k^th^ chunk, l: l^th^ window. The resultant moving window point set (P_MW_) and corresponding reference window point set (P_RW_) are mathematically represented by Equation (8).

8 $${P}_{MW}=\sum _{i=1}^{t}\sum _{j=1}^{4}\sum _{k=1}^{4}\sum _{l=1}^{4}M{W}_{ijkl}(1,1)\quad \quad \quad \quad {P}_{RW}=\sum _{i=1}^{t}\sum _{j=1}^{4}\sum _{k=1}^{4}\sum _{l=1}^{4}R{W}_{ijkl}(1,1)$$

**Figure 3:**
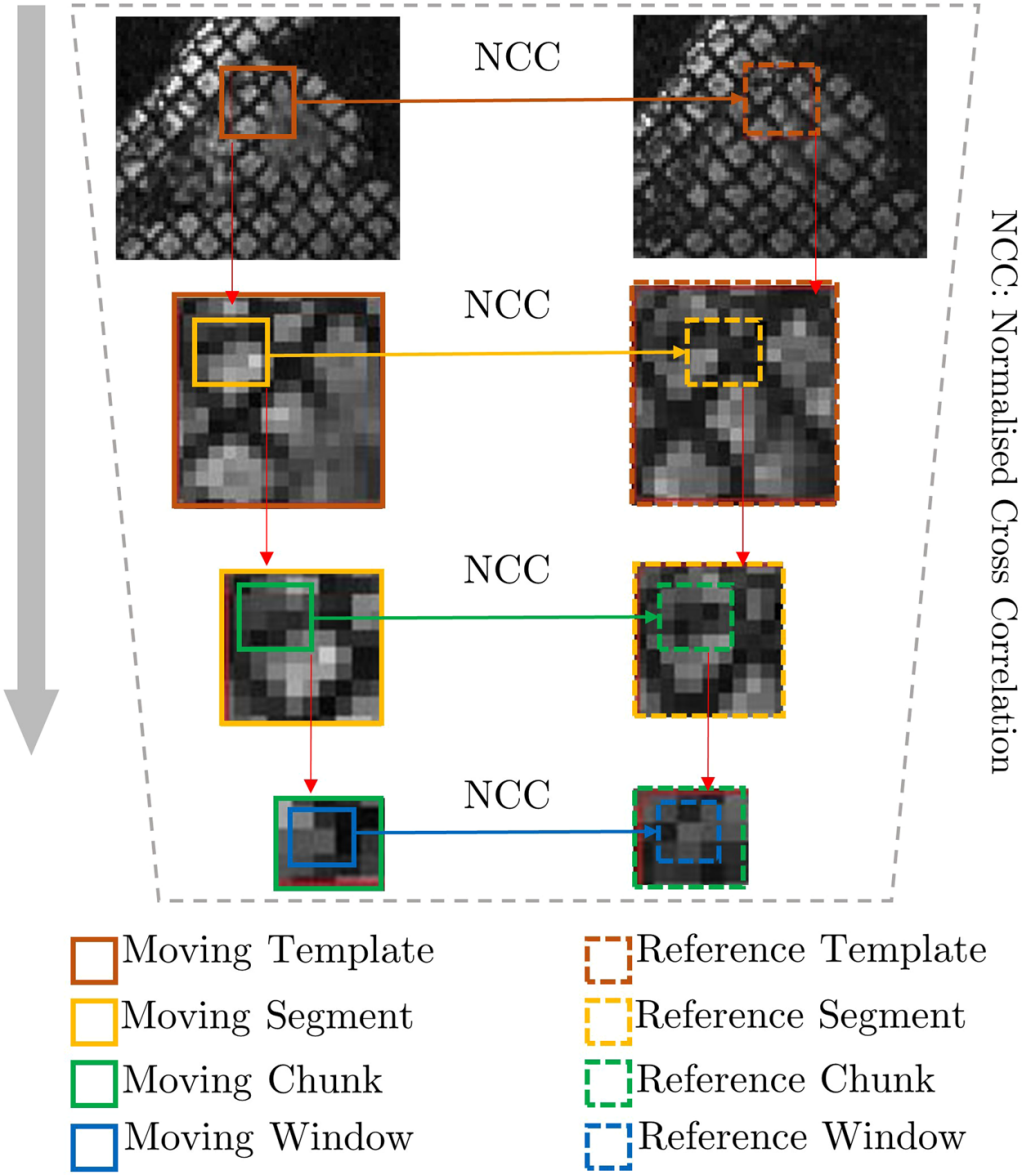
Pictorial representation of Hierarchical Template Matching (HTM) process.

The whole mathematical procedure of hierarchical NCC matching from Equations 2–7 is represented in Fig. 3. P_MW_ and P_RW_ are further used as inputs to estimate geometrical transformation in section 2.3.

### Geometric Transformation of Moving Points into Reference Points

All the moving points are transformed into reference points using landmark-based Local Weighted Mean (LWM) radial basis function. In moving image, landmarks are N moving control points (X_i_, Y_i_), and in the reference image landmarks are corresponding reference control points (x_i_, y_i_), which are mentioned in Equation (8).

9 $$\{({x}_{i},{y}_{i}),\,({X}_{i},{Y}_{i}):i=1,\ldots ,N\}$$

10 $${X}_{i}={f}_{x}({x}_{i},\,{y}_{i}),\,{Y}_{i}={f}_{y}({x}_{i},\,{y}_{i})$$

Or,

11 $${X}_{i}\approx {f}_{x}({x}_{i},\,{y}_{i}),\,{Y}_{i}\approx {f}_{y}({x}_{i},\,{y}_{i})$$

The measurements are arranged in a surface f(x, y) as per equation (12).

12 $$\{({x}_{i},{y}_{i},{f}_{i}):i=\mathrm{1..}N\}$$

A polynomial (Poly_i_) passing through the measurement (x_i_, y_i_, f_i_) and its (n-1) nearest neighbour control points is calculated. For an arbitrary point (x, y) the weighted mean of all polynomials passing through that point has been calculated. The weight function is mentioned in Equation (13).

13 $$\begin{array}{c}{W}_{i}({\rm{D}})=1-3{D}^{2}+2{D}^{3},0\le D\le 1\\ {W}_{i}({\rm{D}})=0,D > 1\end{array}$$

where $D={[{(x-{x}_{i})}^{2}+{(y-{y}_{i})}^{2}]}^{1/2}/{D}_{n}$ and *D*_*n*_ is the distance between (x_i_, y_i_) and (n-1)^th^ nearest control point. Therefore, if control points have a greater distance than *D*_*n*_ then they will not affect the transformation of that point. Moreover, the derivation of W with respect to D at D = 0 and D = 1 is 0. It ensures that the weighted sum is continuous and smooth at all the points. The transformation function at any arbitrary point (x, y) is defined in Equation (14).

14 $${f}_{(x,y)}=\frac{{\sum }_{i=1}^{N}W\{{[{({\rm{x}}-{{\rm{x}}}_{{\rm{i}}})}^{2}+{({\rm{y}}-{{\rm{y}}}_{{\rm{i}}})}^{2}]}^{1/2}/{{\rm{D}}}_{{\rm{n}}}\}Pol{y}_{i}({\rm{x}},{\rm{y}})}{{\sum }_{i=1}^{N}W\{{[{({\rm{x}}-{{\rm{x}}}_{{\rm{i}}})}^{2}+{({\rm{y}}-{{\rm{y}}}_{{\rm{i}}})}^{2}]}^{1/2}/{{\rm{D}}}_{{\rm{n}}}\}}$$

### Cardiac motion and strain calculation

Cardiac motion is defined as the change in locations of the myocardial points. The cardiac motion function is initialised with the myocardial points of image I_1_, which is the first image of the cardiac cycle recorded at time t. The geometric transformation function tracks the location of these points in image I2, which is the second image of the cardiac cycle at time t + Δt. The entire cardiac cycle is recorded with n images (I_1_, I_2_, I_3_, …, I_n_). Therefore, the geometric transformation has been performed sequentially (I_1_ → I_2_ → … → I_n-1_ → I_n_) with consecutive images of the cardiac cycle. The motion estimation and strain calculation are aligned with literature (Khaled *et al*., 2009; Gao *et al*.^42^).

The displacement gradient is calculated with respect to the initial image I_1_. Equation (15) defines the 2D displacement gradient *∂U*. L is the position vector of the moving image and L_1_ is the same position vector of the initial image I_1_.

15 $$\partial U=[\begin{array}{cc}{U}_{xx} & {U}_{xy}\\ {U}_{yx} & {U}_{yy}\end{array}]=\partial (L-{L}_{1})$$

The deformation gradient F is defined in Equation (16).

16 $$F={(I-\partial U)}^{-1}$$

The circumferential and radial strain during the whole cardiac cycle has been derived from the deformation gradient. Eulerian strain tensor E is defined in Equation (17).

17 $$E=\frac{1}{2}[I-{(F{F}^{T})}^{-1}]$$

**Algorithm 1:**
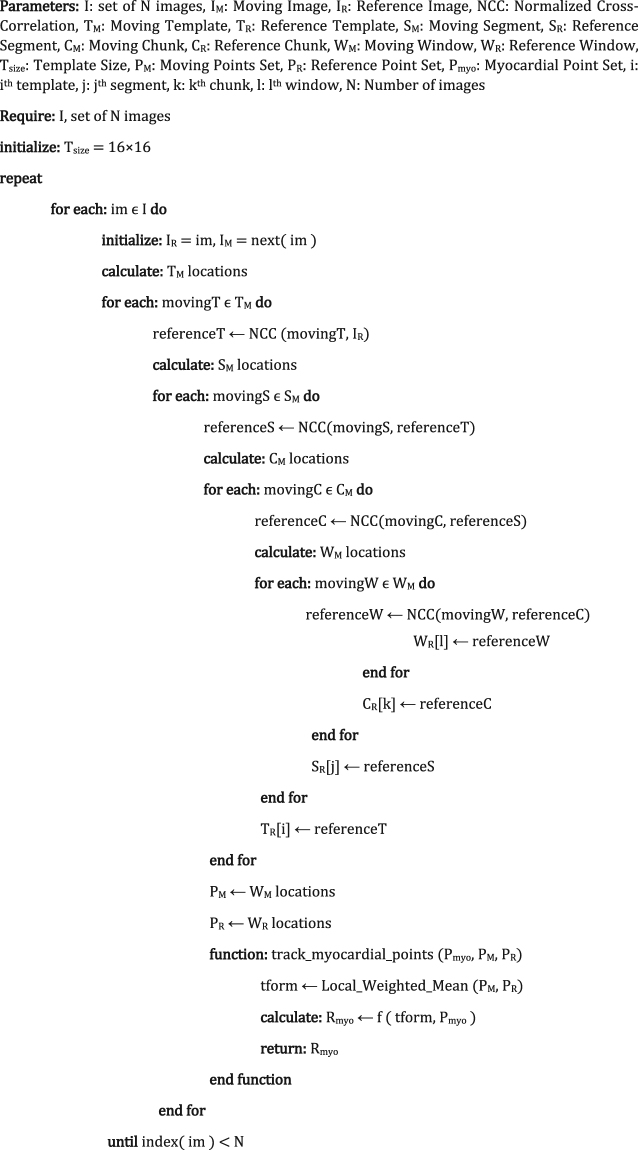
C ardiac Motion Estimation with HTM.

## Results

**Figure 4:**
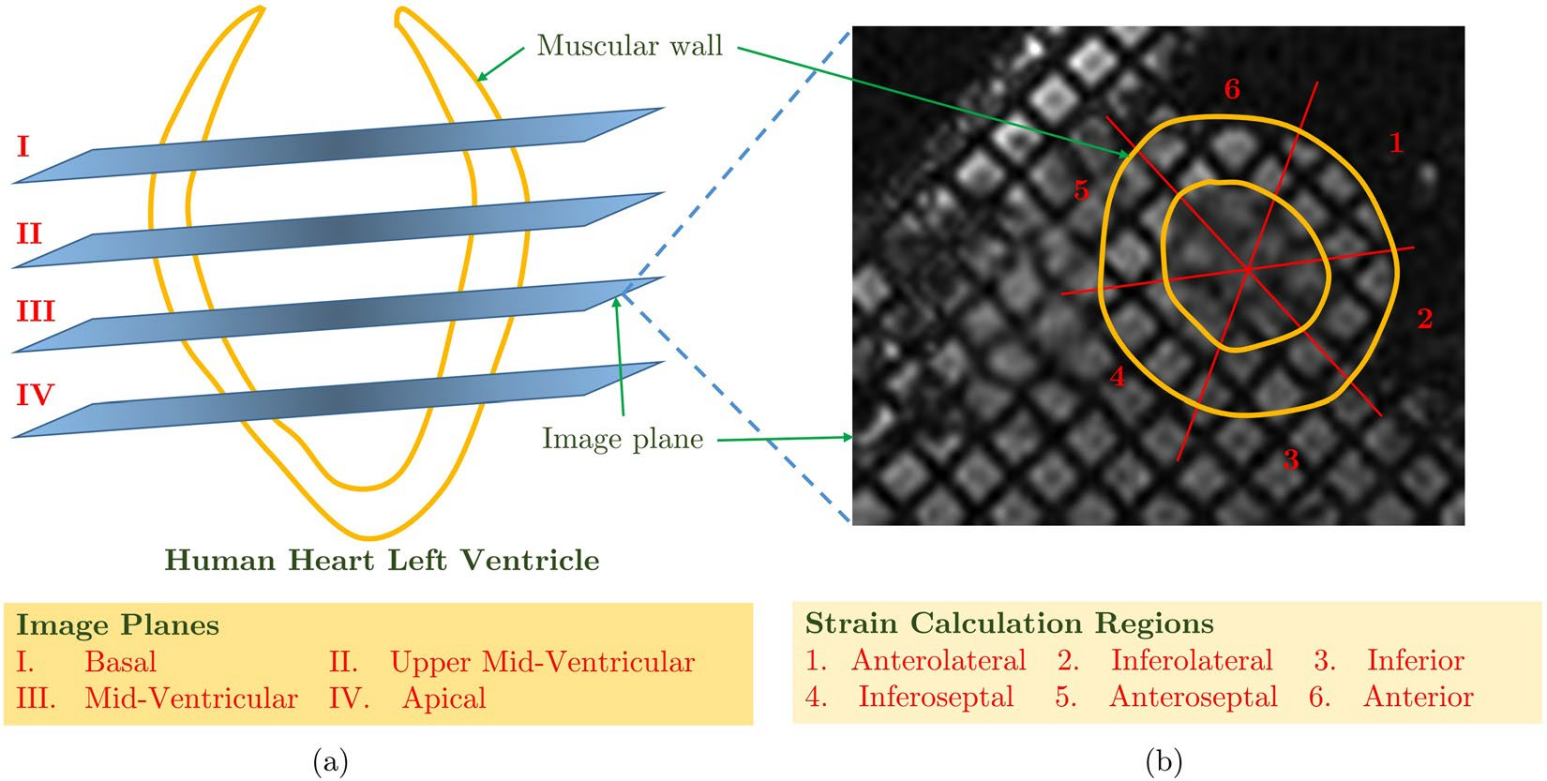
(**a**) Four different LV plane positions during MRI, and (**b**) six different regions of the LV muscular wall for strain calculation.

The proposed, HTM cardiac motion model has been applied to a dataset of 15 healthy subjects which contains 1140 short-axis images. The assessment is performed using Target Registration Error (TRE) using 18 landmarks of the Left Ventricle (LV) wall. The clinical measure of muscles displacement has achieved an accuracy of less than half a millimetre of cardiac muscle. HTM significantly (p < 0.001) reduces the registration error 30.97% compared to the registration error obtained from FFD for 1080 image pairs. The Root Mean Square Error (RMSE) at four different LV level (basal, upper mid-ventricular, mid-ventricular and apical; Fig. 4) is calculated to show the accuracy of the HTM model all over the LV wall muscles. Another important clinical measure is the ventricular wall strain during the cardiac cycle. Circumferential and radial strain values using a data of healthy subject have been estimated in six regions (Figs 4 and 8) of LV myocardial wall. The details of the data set and the validation method are mentioned in section 3.1, and the quantitative results are reported in section 3.2, 3.3, and 3.4.

### Dataset and Validation

The data has been obtained using two different scanners to investigate the robustness of our method. (i) 1.5 T Optima MR450w of GE MRI scanner from University Hospital Coventry and Warwickshire (UHCW), Coventry, UK; with pixel size 1.48 × 1.48 mm (ii) 3 T SKYRA of SIEMENS MRI scanner from Royal Brompton Hospital, London, UK; with pixel size 1.69 × 1.69 mm. Biomedical and Scientific Research Ethics Committee (BSREC) approval (REGO-2016-1865) has been obtained to conduct the study on anonymised human heart data. ECG-gated cardiac tagged MRI has been recorded with proper breath holds and Steady-State Free Precision (SSFP). As mentioned in Fig. 4, the human LV images have been captured at four different levels: basal (top of LV), upper mid-ventricular, mid-ventricular and apical (bottom of LV). The cardiac cycle has been captured with 19 sequential phases of short-axis images (SAX). The initial phases represent diastole, and the later phases represent systole.

The geometric transformation is the core part of the model, and it has been quantitatively validated using two approaches: (i) Target Registration Error (TRE), and (ii) comparison with the benchmark model FFD. The first approach, TRE is adapted from literature^10,27,42^ which is reported as a most important accuracy measure^42^. It calculates the RMSE between corresponding known landmarks which are not used while calculating transformation. The second method is a comparison with improved FFD model^26^, which is an extension of classical FFD model^21^. FFD is reviewed with least RMSE error compared to existing methods^23^. Non-rigid registration results are obtained using latest FFD source code in order to compare the RMSE of HTM and FFD. In this study, the following values of FFD are used: (i) similarity measure is normalised mutual information (ii) regularisation function is bending energy (BE) (iii) value of BE is 0.001 (iv) the linear energy term is 0.01 (v) control point grid levels are 3. The accuracy of the FFD model could be improved if the distances between control points are reduced^21^. Therefore, initial, second, and final level has 4 × 4, 2 × 2 and 1 × 1 control point spacing respectively.

### Target Registration Error – RMSE of HTM

**Figure 5:**
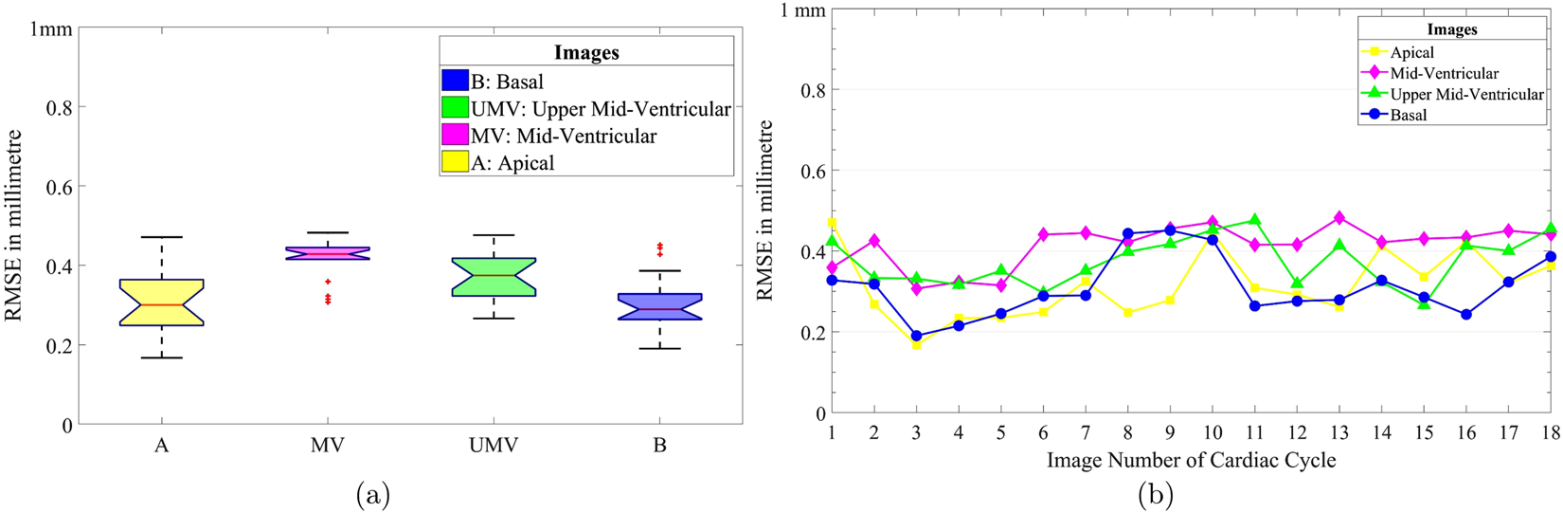
(**a**) TRE of basal, upper mid-ventricular, mid-ventricular, and apical level of LV. (**b**) Separate TRE of all image pairs of cardiac cycle at all four levels.

The tagged MRI SAX images have known landmarks distributed over all six regions. These landmarks are tracked during the cardiac cycle to calculate RMSE in millimetre (mm), which provides a clinical measure of LV wall displacement. The RMSE is applied on pairs of actual landmark locations and tracked landmark locations, which provides RMSE of differences between actual and tracked landmarks. As mentioned in Fig. 5(a), the basal level mean error is 0.3101 ± 0.0053 mm, the upper mid-ventricular level mean error is 0.3743 ± 0.0035 mm, the mid-ventricular error is 0.4140 ± 0.0026 mm, and the apical level mean error is 0.3179 ± 0.0065 mm. The reported mean error with HTM is less than half a millimetre in all cases. The results clearly show that HTM is capable of providing the desired accuracy during image registration without using tunable parameters as used in FFD. Moreover, a significant reduction of RMSE is also mentioned in section 3.3.

### Comparison of HTM and FFD

**Figure 6:**
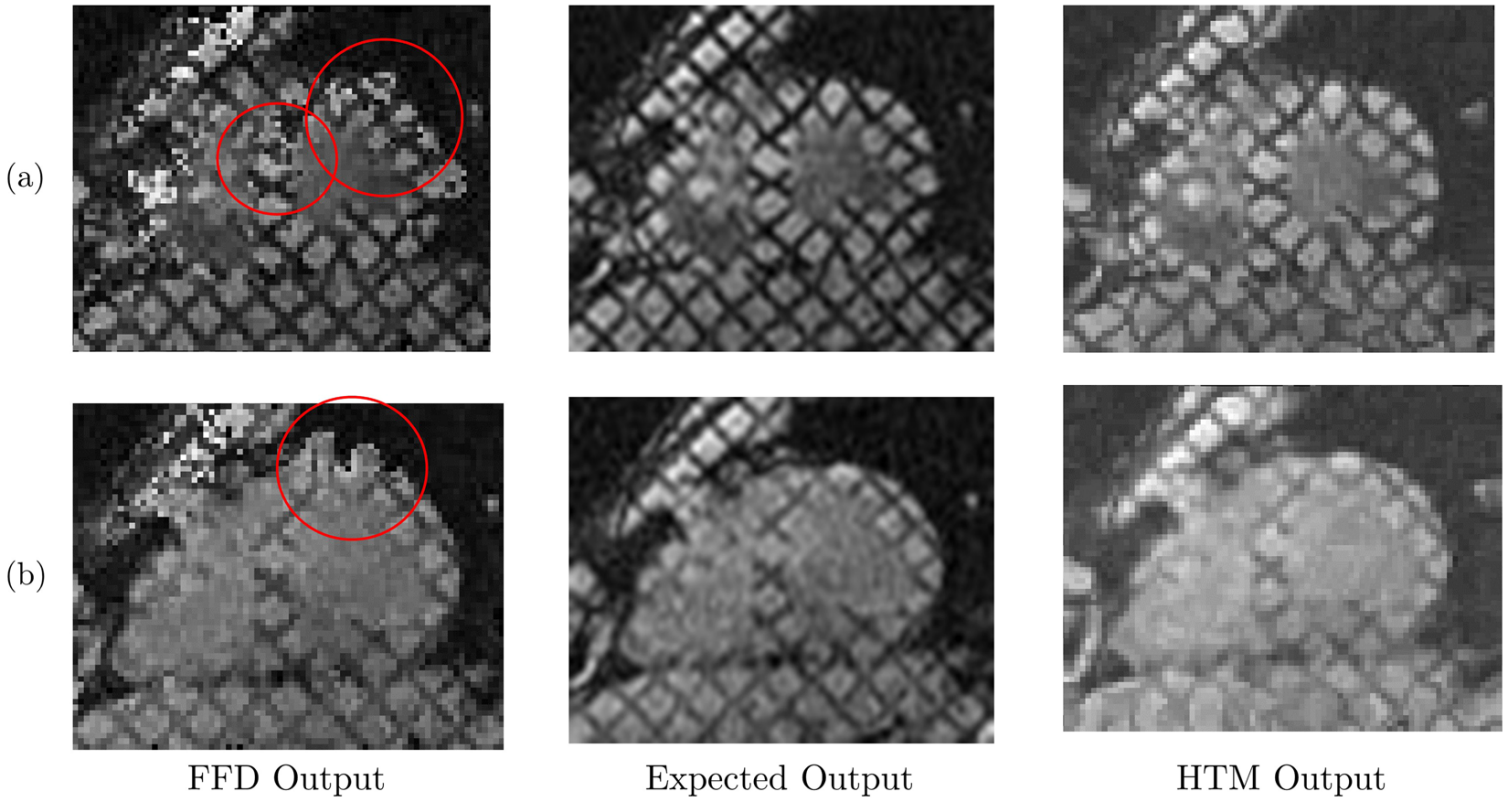
Comparison of output images using FFD and HTM. Middle image (in both cases (**a**) and (**b**)) is the expected output image. Left and right images are respectively FFD output image and HTM output image. Red circles highlight inaccurately transformed muscles of LV wall.

**Figure 7:**
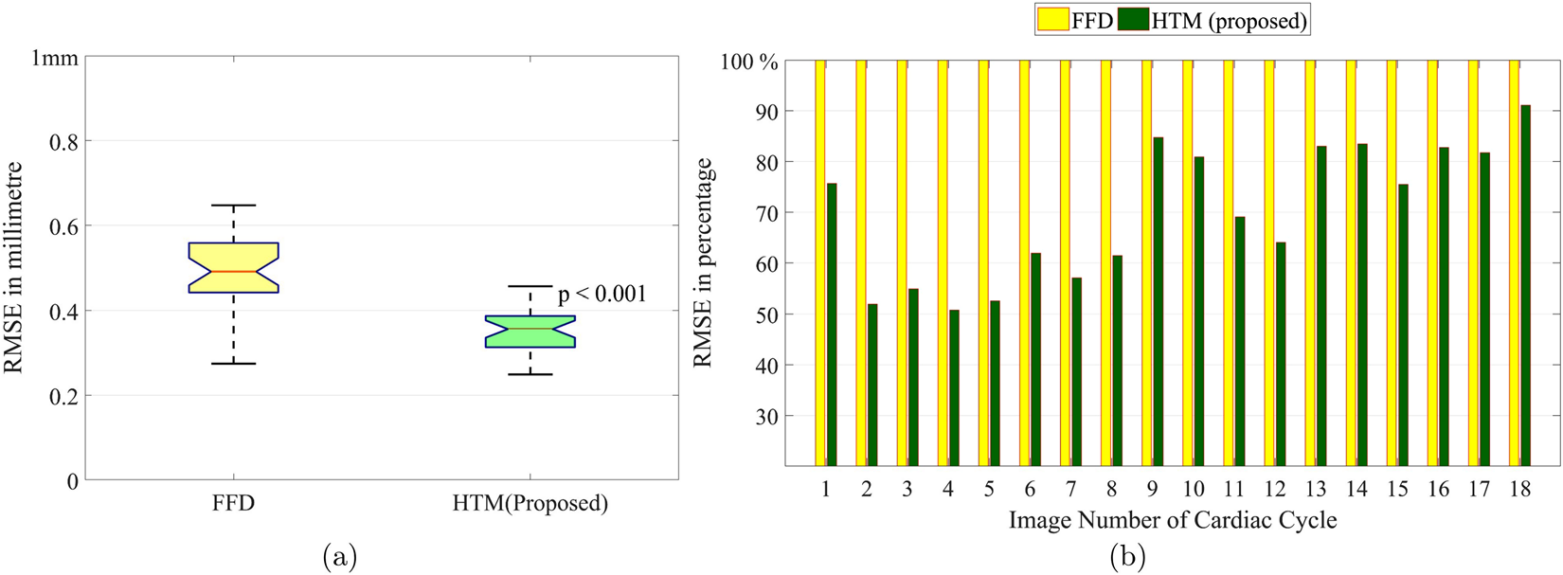
(**a**) Mean RMSE comparison using 1080 SAX image pairs from 15 normal subjects. HTM has significantly reduced error with p < 0.001 for a paired-sample t-test. (**b**) Percentage error comparison during all phases of four different cardiac cycles. The yellow bar is FFD error which is the base (100%), and accordingly, the green bar is HTM error in percentage. The difference of heights of yellow and green bars is percentage error reduction using HTM.

The transformed images, which are obtained using FFD and HTM, have been compared with the expected results as shown in Fig. 6. It is observed that the transformed images produced using HTM provide better results as compared to the transformed images obtained from FFD. FFD failed to transform muscles of Anterior, Anterolateral and Anteroseptal regions of the myocardial wall (red circles). From a paired sample t-test, it is observed that HTM significantly (p < 0.001) reduces TRE compared to FFD. The significant reduction is depicted in Fig. 7(a). Figure 7(b) shows the percentage improvement in accuracy obtained from HTM in comparison with FFD. The FFD error (yellow bar) is considered as a base, i.e. 100% and with respect to that, the HTM error percentage is shown using the green bars. The difference in the height of yellow and green bars shows the percentage improvement with HTM model. The comparative analysis shows that HTM provides almost 50% improvement in 20% of the images, and more than 30% improvement in 50% of the images.

### Strain analysis

**Figure 8:**
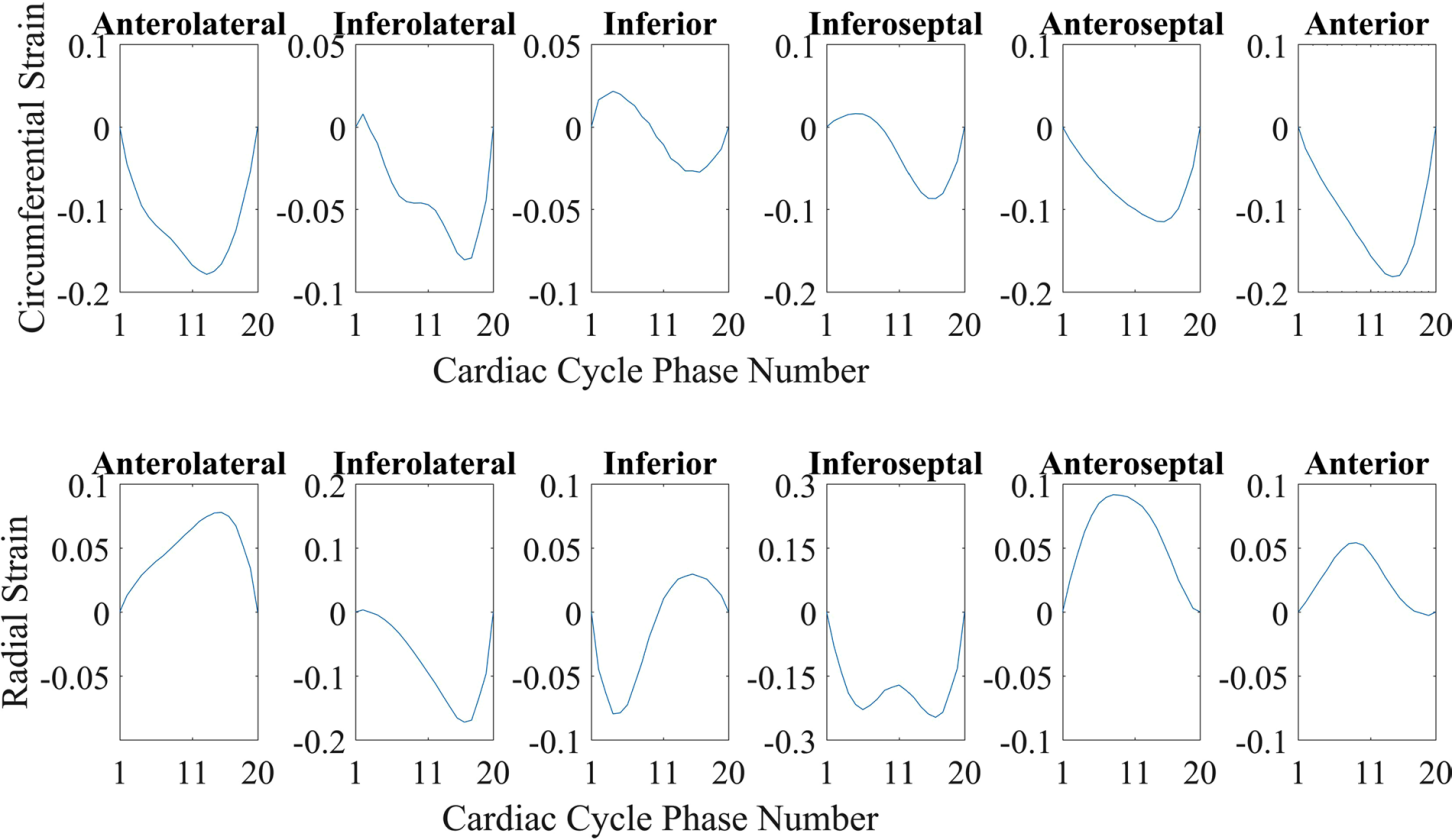
Circumferential and radial strain values over the cardiac cycle. All six regions have different behaviour and strain pattern.

Circumferential and radial strain values of a healthy volunteer have been calculated during the cardiac cycle with a promising accuracy of less than half a millimetre of cardiac muscle. The mechanics of the heart is not uniform for all the heart muscles. Therefore, myocardial muscles have different strain values in different regions. SAX images have been divided into six different regions as per American Heart Association (AHA) model. As shown in Fig. 4(b), the six regions are Anterolateral, Inferolateral, Inferior, Inferoseptal, Anteroseptal, Anterior. Strain values for each of the regions are separately plotted in Fig. 8. Circumferential strain value curves show that they are increasing gradually in the beginning and reducing faster in the later part. It is because of the reduced filling of the blood in LV followed by contraction and rapid ejection. The strain values are qualitatively similar and within the typical limits mentioned in the literature for human LV^43^.

## Discussion

A novel patient-specific model HTM is proposed to estimate cardiac motion and to calculate myocardial strain values using human heart MRI. The model is based on the local weighted-mean geometric transformation of image points, which are obtained using robust hierarchical template matching. The HTM has five advantages over existing literature methods. First, HTM is independent of tunable parameters and tag MRI intersection points, which makes it significantly accurate than benchmark model-FFD. Second, radial strain calculated by FFD is affected by regulariation term^27^, whereas HTM does not use regularisation term, and therefore, the calculated strain values are not affected by regularisation function. Third, the control points are dense and uniform all over the image, therefore, HTM can provide better accuracy compared to the thin-plate spline and multiquadric based models^35^. Fourth, HARP is natively two dimensional, but HTM can be extended to the higher dimensions^33,34^ which provides flexibility to improve the cardiac analysis by three-dimensional extension of HTM. Fifth, the transformation function of HTM does not need a solution of a system of equations which makes it mathematically and computationally simplified compared to the elaborate spline procedure which is used in FFD^33,35,37^. The transformation function of HTM is locally sensitive, which can transform smaller areas of the image precisely, and therefore the resultant muscles tracking can be performed with an accuracy of less than half a millimetre of cardiac muscle. The calculated circumferential and radial strain of six different LV regions are similar to literature^43^.

The patient-specific muscular strain value-based disease diagnosis and prognosis is still in its clinical infancy^7^, but researchers have reported possible applications for CVD patients^18^. For example, patients with the ischemic cardiomyopathy could be benefitted by end-systolic strain-based diagnosis^13^. The prognosis of patients with cardiomyopathy might be improved by observing the difference in strain values^14^. However, the strain value based prognosis and therapy in cardiomyopathy is under research, and therefore, the future benefit from derived muscular strain values needs to be tested on a large group of patients, and the generalized strain range might be useful for clinical decisions. Cardiac Resynchronization Therapy (CRT) with specialised pacemakers have been used in heart failure patients^17^. The cardiac muscles in these patients do not shorten or lengthen appropriately during heart function. Pacemaker leads (wire-end) stimulate various parts of the heart, right and left ventricles, to mimic the finely tuned rhythmic movement of the heart. Identifying the optimum position for the pacemaker leads can be challenging, and not routinely performed. The positions in which the leads settle are crucial for rhythmic heart movement. Strain values can be calculated for various potential lead positions, and the most efficient could be used to ensure leads are positioned as close to the identified position as possible. The identified position could be reached transvenously or epicardially via minimal access techniques. Strain values could be instead of currently used ventricular ejection fraction to guide timing of intervention in patients with valvular disease^15^. In chemotherapy patients suffering from cardiotoxicity (heart becomes weaker to pump blood) could be treated more efficiently with proper strain value-based cardiac assessment^16^ which may help to reduce the mortality with chemotherapy.

The limitation of the proposed method is generating an ill-conditioned polynomial with a fewer (less than 6) number of points. However, this limitation can be eliminated by selecting a sufficient number of local points (recommended 12). The proposed method may not give promising results for the images which are affected by high sensor noise of MRI scanner, but standard quality scanner with a trained operator can eliminate this limitation by developing better quality images.

The future work is the extension of HTM model for three dimensions which will incorporate vertical movement of the heart to provide us with the improved clinical measures. The strain values could be incorporated in biomechanics^44–48^ model with tissue properties constraint for designing future therapeutic interventions.

## Conclusion

In this paper, a novel HTM algorithm is proposed for cardiac motion estimation which is independent of tunable parameters, regularisation function, tag MRI intersection points, and extendable to higher dimensions. The reported results have shown the promising accuracy of less than half a millimetre of cardiac muscles. The calculation of cardiac strain values is performed for a detailed local assessment of cardiac muscles. The impact of the outcome has been focused on the betterment of patient-specific cardiac disease diagnosis and prognosis.

### Data availability

The dataset generated during the current study and code are available from the corresponding author on reasonable request. The original MRI scans can be accessible from the authors SKP and SKB as per NHS rules and regulations.
